# A New Essential Oil from the Leaves of the Endemic Andean Species *Gynoxys miniphylla* Cuatrec. (Asteraceae): Chemical and Enantioselective Analyses

**DOI:** 10.3390/plants11030398

**Published:** 2022-01-31

**Authors:** Omar Malagón, Patricio Cartuche, Angel Montaño, Nixon Cumbicus, Gianluca Gilardoni

**Affiliations:** 1 Departamento de Química, Universidad Técnica Particular de Loja (UTPL), Calle Marcelino Champagnat s/n, Loja 110107, Ecuador; omalagon@utpl.edu.ec (O.M.); apcartuche1@utpl.edu.ec (P.C.); aamontano1@utpl.edu.ec (A.M.); 2 Departamento de Ciencias Biológicas, Universidad Técnica Particular de Loja (UTPL), Calle Marcelino Champagnat s/n, Loja 110107, Ecuador; nlcumbicus@utpl.edu.ec

**Keywords:** *Gynoxys miniphylla*, Asteraceae, essential oil, enantioselective analysis, Ecuador

## Abstract

A previously uninvestigated essential oil (EO) was distilled from *Gynoxys miniphylla* Cuatrec. (Asteraceae) and submitted to chemical and enantioselective analyses. The qualitative and quantitative analyses were conducted by GC-MS and GC-FID, over two orthogonal columns (5%-phenyl-methylpolysiloxane and polyethylene glycol stationary phases). Major constituents (≥2%) were, on both columns, respectively, as follows: α-phellandrene (16.1–17.2%), α-pinene (14.0–15.0%), germacrene D (13.3–14.8%), *trans*-myrtanol acetate (8.80%), δ-cadinene (4.2–4.6%), β-phellandrene (3.3–2.8%), (*E*)-β-caryophyllene (3.1–2.0%), *o*-cymene (2.4%), α-cadinol (2.3–2.6%), and α-humulene (1.7–2.0%). All the quantified compounds corresponded to 93.5–97.3% by weight of the whole essential oil, with monoterpenes counting for 53.8–55.6% of the total, and sesquiterpenes for 38.5–41.4%. For what concerns the enantioselective analyses, the chiral components were investigated through a β-cyclodextrin-based enantioselective column (2,3-diethyl-6-*tert*-butyldimethylsilyl-β-cyclodextrin). A total of six chiral metabolites were analysed and the respective enantiomeric excess calculated as follows: (1*S*,5*S*)-(−)-α-pinene (98.2%), (1*S*,5*S*)-(−)-β-pinene (11.9%), (1*R*,5*R*)-(+)-sabinene (14.0%), (*R*)-(−)-α-phellandrene (100.0%), (*R*)-(−)-β-phellandrene (100.0%), and (*S*)-(−)-germacrene D (95.5%). According to the chemical composition and enantiomeric distribution of major compounds, this EO can be considered promising as a cholinergic, antiviral and, probably, analgesic product.

## 1. Introduction

Since the beginning of 19th century, plants have been thoroughly investigated as sources of new active principles. Nowadays, most of the European vegetal species have been deeply studied and their phytochemical profiles are well known. Therefore, the search for new natural products moved from temperate to tropical countries, where an incredible biodiversity, together with historical and logistical reasons, make the phytochemical investigation more difficult but also more suitable of interesting findings. Seventeen countries have been notably defined as “megadiverse” by the UN Environment Programme, for possessing two thirds of all non-fish vertebrate and three quarters of all higher plant species in the world [1]. Among these countries we can mention Ecuador, whose flora has been very little investigated so far from the chemical point of view [2,3]. For this reason, some of the authors (O.M. and G.G.) have been working for more than 20 years in the purification and structure elucidation of secondary metabolites, isolated from species of the Ecuadorian biodiversity [4,5,6,7,8,9,10]. During the last few years, our attention has been especially drawn by the chemistry of the essential oils (EOs), including the enantiomeric distribution of their chiral components [11,12,13,14,15,16]. Since volatile fractions are not only biologically interesting mixtures but also economically attractive products, we have recently decided to systematically investigate the genus *Gynoxys*, belonging to the family Asteraceae, with a special focus on the EOs. In fact, they find nowadays many commercial applications as flavours and flagrances, for example in foods, pharmaceuticals, cosmetics, perfumery, aromatherapy and household detergents.

The genus *Gynoxys* counts for 162 accepted species and is spread through the Andean region, from Colombia to Bolivia, with a few species also observed in Venezuela [17]. Of all these taxa, 33 have been described in Ecuador, most of them (23) being endemic [18]. According to literature, only a few species have been phytochemically studied so far. On one hand, *G. acostae*, *G. buxifolia*, *G. nitida*, *G. verrucosa*, *G. oleifolia*, *G. sancto antonii*, *G. dielsiana*, and *G. psilophylla* have been studied for their non-volatile constituents, with furanoeremophilanes and sesquiterpene lactones as typical secondary metabolites [19,20,21,22,23,24,25]. On the other hand, only from *G. meridana* and *G. verrucosa*, two EOs, dominated by sesquiterpenes, have been described [26,27].

For what concerns *Gynoxys miniphylla* Cuatrec., it is an Andean endemic shrub, apparently only present in Ecuador, where it grows between 3500 and 4000 m above the sea level. Azuay, Chimborazo, Loja and Morona-Santiago are the provinces where this species has been observed [17,18]. However, no ethnobotanical use is known for this plant. To the best of the authors’ knowledge, this is the first chemical and enantioselective description of an EO distilled from *G. miniphylla*.

## 2. Results

### 2.1. Distillation of the Essential Oil

The fresh leaves of *G. miniphylla* produced, after a 4 h distillation, 2.05 g of a yellow spicy essential oil, that spontaneously separated from the aqueous phase. The yield corresponded to 0.02% by weight, with a density of 0.819 g/cm^3^.

### 2.2. Qualitative and Quantitative Analyses

**Figure 1:**
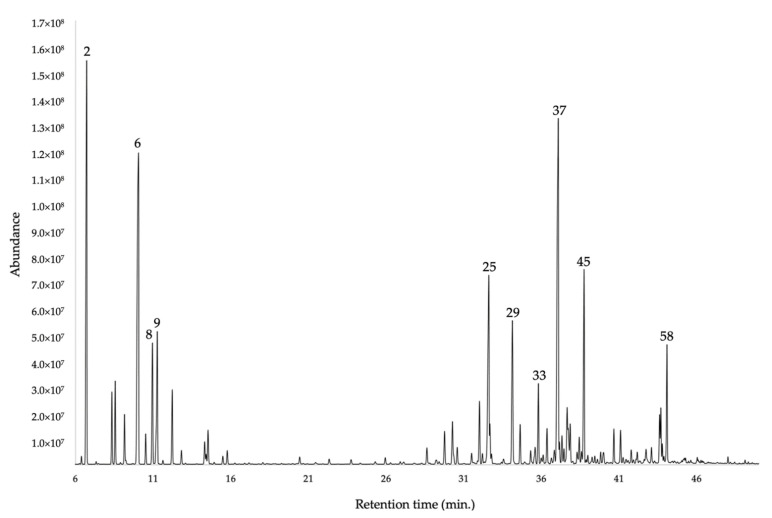
GC-MS chromatogram of *G. miniphylla* EO obtained with a 5%-phenyl-methylpolysiloxane capillary column. The numbers correspond to the main components (≥2% with at least one column).

**Figure 2:**
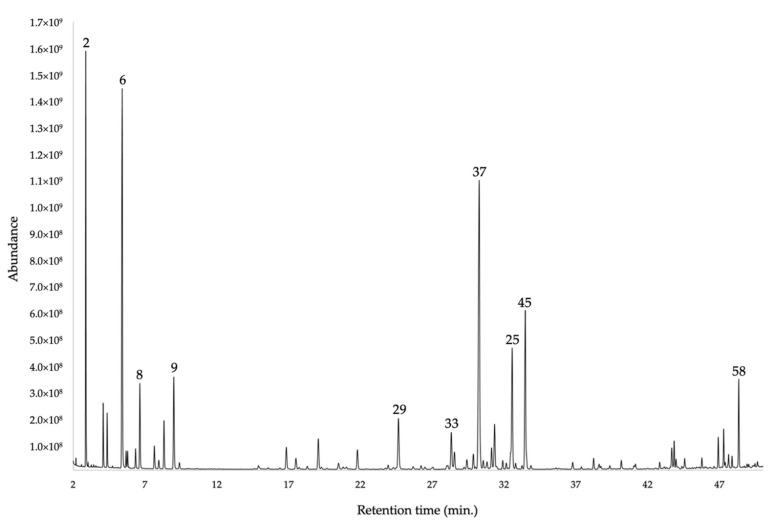
GC-MS chromatogram of *G. miniphylla* EO, obtained with a polyethylene glycol capillary column. The numbers correspond to the main components (≥2% with at least one column).

The qualitative chemical analysis of the EO resulted in the identification of 59 compounds, all quantified with at least one column. The total quantification of the components corresponded to 93.5–97.3% by weight of the whole EO, through a non-polar (5%-phenyl-methylpolysiloxane) and a polar (polyethylene glycol) stationary phase, respectively. The main constituents (≥2%) were α-phellandrene (16.1–17.2%), α-pinene (14.0–15.0%), germacrene D (13.3–14.8%), *trans*-myrtanol acetate (8.80%), δ-cadinene (4.2–4.6%), β-phellandrene (3.3–2.8%), (*E*)-β-caryophyllene (3.1–2.0%), *o*-cymene (2.4% by both columns), α-cadinol (2.3–2.6%), and α-humulene (1.7–2.0%). Quantitatively, the monoterpene and the sesquiterpene fractions (53.8–55.6% and 38.5–41.4%, respectively) were almost comparable, with a slight excess (about 10%) of monoterpenes. However, the number of sesquiterpenes is quite greater than the one of monoterpenes, as it appears in Figure 1 and Figure 2. The quantitative analysis was carried out in four repetitions for each column, and the results were expressed as mean values and standard deviations. All the qualitative and quantitative results are reported in Table 1. The gas-chromatography (GC) profiles are represented in Figure 1 and Figure 2.

### 2.3. Enantioselective Analysis

**Figure 3:**
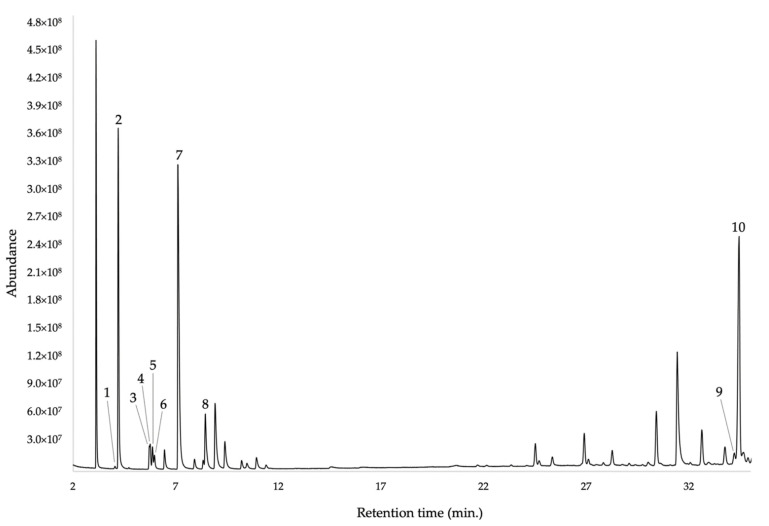
GC-MS chromatogram of *G. miniphylla* EO obtained with a 2,3-diethyl-6-*tert*-butyldimethylsilyl-β-cyclodextrin capillary column. 1: (1*R*,5*R*)-(+)-α-pinene; 2: (1*S*,5*S*)-(−)-α-pinene; 3: (1*R*,5*R*)-(+)-β-pinene; 4: (1*S*,5*S*)-(−)-β-pinene; 5: (1*R*,5*R*)-(+)-sabinene; 6: (1*S*,5*S*)-(−)-sabinene; 7: (*R*)-(−)-α-phellandrene; 8: (*R*)-(−)-β-phellandrene; 9: (*R*)-(+)-germacrene D; 10: (*S*)-(−)-germacrene D.

The enantioselective analysis of the EO was carried out on a 2,3-diethyl-6-*tert*-butyldimethylsilyl-β-cyclodextrin based capillary column. A total of 6 chiral terpenes were analysed, most of them being present as enantiomeric pairs. On the other hand, (*R*)-(−)-α-phellandrene and (*R*)-(−)-β-phellandrene resulted enantiomerically pure, whereas (1*S*,5*S*)-(−)-α-pinene and (*S*)-(−)-germacrene D presented an enantiomeric excess >95%. All the results from the enantioselective analysis are reported in Table 2, and the GC profile in Figure 3.

## 3. Discussion

### 3.1. The EOs of Genus Gynoxys

Despite very few studies having been published on genus *Gynoxys*, especially for what concerns EOs, the following two species have been described for their volatile fractions: *G. meridana* and *G. verrucosa* [26,27]. On one hand, the main components of *G. meridana* EO (>2%) were: γ-curcumene (31.9%), β-pinene (9.5%), α-phellandrene (7.1%), α-pinene (5.7%), valencene (3.8%), and ar-curcumene (2.7%). On the other hand, the major constituents of *G. verrucosa* EO were: α-zingiberene (45.6%), α-amorphene (11.1%), *p*-cymene (15.2%), α-phellandrene (11.7%), thymol methyl ether (3.4%), and (*E*)-β-cariophyllene (3.2%). The comparison of these two EOs with the one from *G. miniphylla* demonstrated that they are all characterized by both an important monoterpene and sesquiterpene fractions, where the number of sesquiterpenes is clearly prevalent, even when the monoterpenes are quantitatively majoritarian (e.g., *G. miniphylla*). The preliminary and still unpublished data so far available effectively demonstrate that the EOs from this genus are tendentially dominated by sesquiterpenes.

### 3.2. Biological Activities of the Main Components

In the EO from the leaves of *G. miniphylla*, the sum of three common terpenes counts for about 50% of the whole amount. According to the elution order, they are α-pinene, α-phellandrene, and germacrene D, each corresponding to more than 15% of the total EO mass. Since the biological properties of the EOs can be partially deduced by the activities of their major compounds, we conducted a short review of the three main terpenes. For what concerns α-pinene, this very common monoterpene is known to possess a wide range of biological activities. It is anti-inflammatory, a human bronchodilator, antibacterial against methicillin-resistant *Staphylococcus aureus* (MRSA), and antifungal against *Cryptococcus neoformans* and *Candida albicans* [43,44,45,46]. Furthermore, α-pinene manifested an interesting activity against the promastigotes of *Leishmania amazonensis* and the larvae of *Anopholes subpictus* (a vector of malaria), *Aedes albopictus* (a vector of dengue), and *Culex tritaeniorhynchus* (vector of Japanese encephalitis) [47,48]. Nevertheless, we agree with some authors that consider the inhibition activity of acetylcholinesterase (AChE) to be the most important property of α-pinene. This activity probably explains, to a great extent, the strong AChE inhibition capacity observed for many monoterpene-based EOs [49,50]. In some previous studies, we could personally observe that this activity is often selectively stronger versus butyrylcholinesterase (BChE) than AChE [51,52]. Many other biological activities have been described for α-pinene, including a very peculiar in vivo anxiolytic effect by inhalation [53,54,55]. Another important component is α-phellandrene. Like α-pinene, also α-phellandrene is one of the most common monoterpenes in EOs, despite its biological properties are less studied than those of the previous metabolite. The most interesting property of α-phellandrene is probably the in vivo antinociceptive activity in rodents [56]. A subsequent study confirmed this activity, by observing an antihyperalgesic action in a neuropathic pain model [57]. Furthermore, α-phellandrene, that apparently does not exert any interesting in vitro anti-microbial action, enhanced the macrophage phagocytosis and the activity of killer cells. This property could result in an in vivo increased immune reaction to pathogenic agents [58]. Additionally, α-phellandrene induced a DNA damage in murine leukaemia cells, also affecting their DNA-repairing capacity in an in vitro study [59,60]. Finally, some literature about germacrene D will be analysed. The main biological property of this sesquiterpene is to interact with specific antennal receptors, located in the moths’ olfactory neurons of some species from genera *Heliothis* and *Helicoverpa*. The effect of this pheromone-like interaction is to increase the attraction and oviposition in these insects [61,62,63]. Anyway, the property of some EO components to act as insect pheromones is well described in literature and quite common in nature [64].

### 3.3. Biological Properties of the Main Enantiomers

The three major constituents α-pinene, α-phellandrene, and germacrene D were among the chiral metabolites, that could be enantioselectively analysed in the present study. Since different enantiomers are notoriously characterised by different biological activities, the enantiomeric composition of an EO must be investigated, in order to get a comprehensive information about its potential properties. In fact, as described in the previous section, bicyclic monoterpenes are important inhibitors of AChE, but the two enantiomers are sometimes characterized by different activities. However, in the case of α-pinene, both enantiomeric forms practically present the same inhibition capacity [50]. Nevertheless, other biological activities of α-pinene are influenced by stereochemistry. Whereas the laevorotatory isomer is active against the infectious bronchitis virus (IBV), the dextrorotatory form (minority in *G. minyphilla* EO) is the most effective antifungal enantiomer against *C. albicans*, *C. neoformans*, and *Rhizopus oryzae* and the strongest antibacterial isomer versus MRSA. Similarly, in the cytotoxicity of α-pinene versus mouse peritoneal macrophages, the dextrorotatory isomer is the most active form [46]. On the other hand, no information has been found in literature about the enantiomerically based properties of α-phellandrene. For what concerns germacrene D, the laevorotatory isomer, dominant in our EO, appeared to be ten times more active than the dextrorotatory form in the previously described pheromone-like activity [62,63]. According to the facts discussed in this section, the frequent lack of enantioselective analysis in the EO studies can explain the discrepancies, often observed in the literature, with respect to the bioactivity data.

## 4. Materials and Methods

### 4.1. GC and GC-MS Analyses

The chemical and enantioselective analyses of *G. miniphylla* EO were carried out with a gas chromatography-mass spectrometry (GC-MS) equipment, consisting of a Trace 1310 gas chromatograph, coupled to a simple quadrupole mass spectrometry detector, model ISQ 7000 (Thermo Fisher Scientific, Walthan, MA, USA). Additionally, a common flame ionization detector (FID) complemented the same instrument. The mass spectrometer was operated in SCAN mode (scan range 35–350 *m*/*z*), with the electron ionization (EI) source set at 70 eV. A non-polar column, based on 5%-phenyl-methylpolysiloxane, and a polar one, based on a polyethylene glycol stationary phase, were applied to both the qualitative and quantitative analyses. The non-polar column was DB-5ms (30 m long, 0.25 mm internal diameter, and 0.25 μm film thickness), whereas the polar one was HP-INNOWax (30 m × 0.25 mm × 0.25 μm), both purchased from Agilent Technology (Santa Clara, CA, USA). The enantioselective analysis was carried out through an enantioselective capillary column, based on 2,3-diethyl-6-*tert*-butyldimethylsilyl-β-cyclodextrin as a chiral selector (25 m × 250 μm internal diameter × 0.25 μm phase thickness), and purchased from Mega, MI, Italy. GC purity grade helium, from Indura, Guayaquil, Ecuador, was used as carrier gas, set at the constant flow of 1 mL/min. For all the GC analysis, the analytical purity grade solvents, the mixture of *n*-alkanes (C_9_–C_25_), and the internal standard (*n*-nonane), were purchased from Sigma-Aldrich (St. Louis, MO, USA). The calibration standard was isopropyl caproate, obtained by synthesis in the authors’ laboratory and purified to 98.8% (GC-FID).

### 4.2. Plant Material

The leaves of *G. miniphylla* were collected on 11 March 2020, on the way to mount Fierro Urku, in the San Lucas parish, Province of Loja, Ecuador. The collection point corresponded to coordinates 3°42′59.6” S and 79°18′51.0” W, at 3388 m above the sea level. The species was identified and classified by one of the authors (N.C.), whereas a botanical specimen was deposited at the herbarium of the Universidad Técnica Particular de Loja, with voucher code HUTPL14301. This investigation was carried out under permission of the Ministry of Environment, Water and Ecological Transition of Ecuador, with MAATE registry number MAE-DNB-CM-2016-0048.

### 4.3. Sample Preparation and EO Distillation

The day after collection, 9.13 Kg of fresh leaves were steam distilled, for 4 h, in a stainless-steel Clevenger-type apparatus, where steam is produced in a separated compartment. The process afforded 2.05 g a yellow essential oil, that spontaneously separated from the aqueous phase. The EO was then dried over anhydrous sodium sulphate and stored in the darkness, at −15 °C, until use. All the GC analyses were conducted by injecting diluted samples, prepared as previously described in the literature [15].

### 4.4. Qualitative Chemical Analysis

The components of the EO were identified by comparing their mass spectrum and linear retention index (LRI) with data present in literature (see Table 1). The LRI was calculated for each constituent according to Van den Dool and Kratz, using the homologous series of *n*-alkanes, from C_9_ to C_25_ [65]. The qualitative analysis was repeated with two orthogonal columns (polar and non-polar), injecting in both 1 μL of the previously described sample in split mode (split ratio 40:1). With the 5%-phenyl-methylpolysiloxane column, the thermal program was as follows: initial temperature 60 °C for 5 min, followed by a first thermal gradient of 2 °C/min until 100 °C, then a second gradient of 3 °C/min until 150 °C, and a third one of 5 °C/min until 200 °C. Finally, a new gradient of 15 °C/min until 250 °C was applied. The final temperature was maintained for 5 min. With the polyethylene glycol column, the thermal program was the same, except for the final temperature that did not exceed 230 °C.

### 4.5. Quantitative Chemical Analysis

The metabolites, previously identified, were subsequently quantified through the same two columns, by means of a flame ionization detector (FID). The quantification was carried out calculating the relative response factor (RRF) of each analyte versus isopropyl caproate, used as a quantification standard. The RRFs were determined according to the combustion enthalpy of each compound, as described in the literature [66]. However, instead of using the isopropyl caproate as internal standard, it was applied for external calibration, whereas *n*-nonane was used for internal normalization [16]. For both columns, the calibration curve afforded a R^2^ > 0.995. The GC methods and conditions were the same as the qualitative analyses. The quantitative results were expressed as mean values and standard deviation, over four repetitions, with each column. The percentage values referred to the weight of each analyte with respect to the mass of the whole essential oil.

### 4.6. Enantioselective Analyses

The enantioselective analyses were conducted by injecting the same previously described samples into the same GC-MS system used for the qualitative analyses. The employed GC method was as follows: the initial temperature was 60 °C for 2 min, followed by a thermal gradient of 2 °C/min until 220 °C, that was maintained for 2 min. The homologous series of *n*-alkanes (C_9_–C_25_) was also injected, in order to calculate the linear retention indices of the stereoisomers. The enantiomers were identified for their MS spectrum and elution order, determined by injection of enantiomerically pure standards.

## 5. Conclusions

The leaves of *G. miniphylla* Cuatrec. produced an EO of monoterpene ad sesquiterpene composition, with the relatively low yield of 0.02% by weight over the fresh plant material. Despite the monoterpene fraction appeared quantitatively dominant, the sesquiterpenes numerically prevailed. According to the chemical composition and enantiomeric distribution of the major compounds, this volatile fraction can be considered promising as a cholinergic, antiviral and, probably, analgesic EO. This hypothesis should be experimentally verified in future studies.

## Figures and Tables

**Table 1:** Chemical analyses of *G. miniphylla* EO obtained with non-polar (5%-phenyl-methylpolysiloxane) and polar (polyethylene glycol) capillary columns.

N.	Compounds	5%-phenyl-methylpolysiloxane				polyethylene glycol				
		IRL ^a^	IRL ^b^	%	σ	IRL ^a^	IRL ^c^	Ref.	%	σ
1	tricyclene	923	921	0.1	0.01	1013	1012	[28]	0.1	0.01
2	α-pinene	931	932	14.0	0.45	1025	1025	[28]	15.0	0.68
3	sabinene	969	969	1.3	0.04	1123	1122	[28]	1.4	0.06
4	β-pinene	974	974	1.7	0.05	1112	1110	[28]	1.8	0.09
5	myrcene	988	988	1.1	0.09	1203	1187	[29]	0.7	0.04
6	α-phellandrene	1007	1002	16.1	0.45	1166	1168	[28]	17.2	0.92
7	α-terpinene	1015	1014	0.5	0.01	1182	1178	[28]	0.5	0.03
8	*o*-cymene	1023	1022	2.4	0.07	1272	1276	[30]	2.4	0.12
9	β-phellandrene	1028	1025	3.3	0.09	1210	1209	[28]	2.8	0.15
10	(*E*)-β-ocimene	1044	1044	1.8	0.07	1254	1250	[28]	1.8	0.10
11	ɣ-terpinene	1055	1054	0.3	0.01	1245	1245	[28]	0.3	0.01
12	camphenilone	1081	1078	0.7	0.02	1177	1456	[31]	0.8	0.04
13	terpinolene	1082	1086	0.2	0.02	1282	1282	[32]	0.3	0.01
14	linalool	1101	1095	0.3	0.01	1565	1556	[33]	0.5	0.46
15	*n*-nonanal	1105	1100	0.5	0.01	1399	1387	[34]	0.3	0.04
16	terpinen-4-ol	1177	1174	0.2	0.01	1607	1601	[28]	0.2	0.02
17	*n*-decanal	1206	1201	0.2	0.01	-	-	-	-	-
18	thymol methyl ether	1228	1232	0.2	0.01	1596	1587	[28]	0.2	0.03
19	2-(*E*)-decenal	1263	1260	0.5	0.04	1645	1640	[28]	trace	-
20	carvacrol	1306	1298	0.5	0.05	2213	2210	[28]	0.4	0.31
21	α-cubebene	1343	1348	0.3	0.01	1447	1460	[28]	0.5	0.05
22	neryl acetate	1361	1359	0.3	0.01	1734	1718	[28]	0.4	0.05
23	α-copaene	1370	1374	1.2	0.03	1476	1491	[28]	1.2	0.13
24	modheph-2-ene	1374	1382	0.2	0.01	1502	1496	[35]	0.3	0.03
25	*trans*-myrtanol acetate	1382	1385	8.8	0.24	1765	1746	[36]	8.8	1.44
26	β-cubebene	1383	1387	0.8	0.02	1526	1542	[28]	0.9	0.11
27	β-elemene	1385	1389	0.2	0.01	-	-	-	-	-
28	α-gurjunene	1399	1409	0.1	0.04	1512	1529	[28]	0.1	0.01
29	(*E*)-β-caryophyllene	1412	1417	3.1	0.08	1577	1578	[37]	2.0	0.39
30	β-copaene	1423	1430	0.4	0.04	1613	1631	[38]	0.2	0.04
31	β-gurjunene	1438	1431	0.2	0.02	-	-	-	-	-
32	aromadendrene	1444	1439	0.3	0.05	1622	1620	[28]	0.1	0.03
33	α-humulene	1448	1452	1.7	0.03	1650	1667	[28]	2.0	0.30
34	allo-aromadendrene	1452	1458	0.1	0.03	1624	1637	[28]	0.2	0.04
35	(*E*)-β-farnesene	1461	1454	0.8	0.01	1655	1664	[28]	1.0	0.14
36	dauca-5,8-diene	1467	1471	0.1	0.01	1644	1654	[39]	0.4	0.16
37	germacrene D	1476	1480	13.3	0.38	1690	1708	[28]	14.8	2.36
38	ar-curcumene	1478	1479	0.5	0.01	1767	1770	[28]	0.8	0.14
39	*cis*-β-guaiene	1482	1492	0.2	0.01	1677	1664	[28]	trace	-
40	*trans*-muurola-4(14),5-diene	1484	1493	0.3	0.01	-	-	-	-	-
41	bicyclogermacrene	1489	1500	1.9	0.06	1714	1730	[28]	1.8	0.26
42	α-muurolene	1493	1500	0.9	0.03	1709	1723	[28]	1.1	0.22
43	(*E*,*E*)-α-farnesene	1503	1505	0.2	0.01	1749	1744	[28]	trace	-
44	δ-amorphene	1510	1511	0.3	0.07	1702	1710	[40]	0.4	0.07
45	δ-cadinene	1514	1522	4.2	0.81	1743	1756	[28]	4.6	1.23
46	β-sesquiphellandrene	1520	1521	0.3	0.01	1759	1771	[28]	trace	-
47	*trans*-cadina-1,4-diene	1527	1533	0.1	0.01	-	-	-	-	-
48	α-cadinene	1531	1537	0.1	0.01	1774	1769	[28]	0.2	0.04
49	(*E*)-nerolidol	1561	1561	0.8	0.01	2057	2053	[34]	1.4	0.62
50	*trans*-sesquisabinene hydrate	1575	1577	0.2	0.02	2128	2092	[28]	0.5	0.16
51	globulol	1588	1590	0.3	0.02	2082	2082	[28]	0.6	0.15
52	viridiflorol	1597	1592	0.2	0.01	2023	2054	[28]	0.5	0.13
53	junenol	1613	1618	0.3	0.01	2052	2052	[41]	trace	-
54	1-*epi*-cubenol	1624	1627	0.3	0.02	2062	2088	[28]	0.4	0.12
55	*epi*-α-cadinol	1640	1638	0.9	0.02	2176	2170	[28]	1.1	0.36
56	*epi*-α-muurolol	1642	1640	1.0	0.02	2192	2186	[28]	1.7	0.52
57	α-muurolol	1645	1644	0.3	0.01	-	-	-	-	-
58	α-cadinol	1654	1652	2.3	0.04	2230	2227	[28]	2.6	1.16
59	cyperotundone	1690	1695	0.1	0.01	-	-	-	-	-
	Monoterpene hydrocarbons			42.8					44.3	
	Oxygenated monoterpenes			11.0					11.3	
	Sesquiterpene hydrocarbons			31.8					32.6	
	Oxygenated sesquiterpenes			6.7					8.8	
	Other compounds			1.2					0.3	
	Total identified			93.5					97.3	

^a^ Calculated linear retention index; ^b^ Linear retention index according to [42]; ^c^ Linear retention index according to reference (Ref.).

**Table 2:** Enantioselective analysis of *G. miniphylla* EO, obtained with a β-cyclodextrin-based capillary column.

N.	Enantiomers	2,3-diethyl-6-*tert*-butyldimethylsilyl-β-cyclodextrin		
		LRI ^1^	ED ^2^ (%)	*ee*^3^ (%)
1	(1*R*,5*R*)-(+)-α-pinene	932	0.9	98.2
2	(1*S*,5*S*)-(–)-α-pinene	938	99.1	
3	(1*R*,5*R*)-(+)-β-pinene	993	44.1	11.9
4	(1*S*,5*S*)-(–)-β-pinene	995	55.9	
5	(1*R*,5*R*)-(+)-sabinene	999	57.0	14.0
6	(1*S*,5*S*)-(–)-sabinene	1001	43.0	
7	(*R*)-(–)-α-phellandrene	1027	100.0	100.0
8	(*R*)-(–)-β-phellandrene	1056	100.0	100.0
9	(*R*)-(+)-germacrene D	1499	4.5	91.0
10	(*S*)-(–)-germacrene D	1504	95.5	

^1^ Linear retention index; ^2^ Enantiomeric distribution; ^3^ Enantiomeric excess.

## Data Availability

Raw data are available from the authors (P.C.).

## References

[1] Megadiverse Countries, UNEP-WCMC. https://www.biodiversitya-z.org/content/megadiverse-countries.

[2] Malagón O., Ramírez J., Andrade J., Morocho V., Armijos C., Gilardoni G. (2016). Phytochemistry and Ethnopharmacology of the Ecuadorian Flora. A Review. Nat. Prod. Commun..

[3] Armijos C., Ramírez J., Salinas M., Vidari G., Suárez A.I. (2021). Pharmacology and Phytochemistry of Ecuadorian Medicinal Plants: An Update and Perspectives. Pharmaceuticals.

[4] Chiriboga X., Gilardoni G., Magnaghi I., Vita Finzi P., Zanoni G., Vidari G. (2003). New Anthracene Derivatives from *Coussarea macrophylla*. J. Nat. Prod..

[5] Quílez A., Berenguer B., Gilardoni G., Souccar C., De Mendonça S., Oliveira L.F.S., Martin-Calero M.J., Vidari G. (2010). Anti- secretory, Anti-inflammatory, and Anti-*Helicobacter pylori* Activities of Several Fractions Isolated from *Piper carpunya* Ruiz & Pav. J. Ethnopharmacol..

[6] Gilardoni G., Tosi S., Mellerio G., Maldonado M.E., Chiriboga X., Vidari G. (2011). Lipophilic Components from the Ecuadorian Plant *Schistocarpha eupatorioides*. Nat. Prod. Commun..

[7] Gilardoni G., Malagon O., Morocho V., Negri R., Tosi S., Guglielminetti M., Vidari G., Vita Finzi P. (2011). Phytochemical Research and Antimicrobial Activity of *Clinopodium nubigenum* Kunth (Kuntze) Raw Extracts. Rev. Bras. Farmacogn..

[8] Gilardoni G., Chiriboga X., Finzi P.V., Vidari G. (2015). New 3,4-Secocycloartane and 3,4-Secodammarane Triterpenes from the Ecuadorian Plant *Coussarea macrophylla*. Chem. Biodivers..

[9] Herrera C., Pérez Y., Morocho V., Armijos C., Malagón O., Brito B., Tacán M., Cartuche L., Gilardoni G. (2018). Preliminary Phytochemical Study of the Ecuadorian Plant *Croton elegans* Kunth. (Euphorbiaceae). J. Chil. Chem. Soc..

[10] Morocho V., Valarezo L.P., Tapia D.A., Cartuche L., Cumbicus N., Gilardoni G. (2021). A Rare Dirhamnosyl Flavonoid and Other Radical-scavenging Metabolites from *Cynophalla mollis* (Kunth) J. Presl and *Colicodendron scabridum* (Kunt) Seem. (Capparaceae) of Ecuador. Chem. Biodivers..

[11] Gilardoni G., Montalván M., Vélez M., Malagón O. (2021). Chemical and Enantioselective Analysis of the Essential Oils from Different Morphological Structures of *Ocotea quixos* (Lam.) Kosterm. Plants.

[12] Calvopiña K., Malagón O., Capetti F., Sgorbini B., Verdugo V., Gilardoni G. (2021). A New Sesquiterpene Essential Oil from the Native Andean Species *Jungia rugosa* Less (Asteraceae): Chemical Analysis, Enantiomeric Evaluation, and Cholinergic Activity. Plants.

[13] Ramírez J., Andrade M.D., Vidari G., Gilardoni G. (2021). Essential Oil and Major Non-Volatile Secondary Metabolites from the Leaves of Amazonian *Piper subscutatum*. Plants.

[14] Espinosa S., Bec N., Larroque C., Ramírez J., Sgorbini B., Bicchi C., Cumbicus N., Gilardoni G. (2021). A Novel Chemical Profile of a Selective In Vitro Cholinergic Essential Oil from *Clinopodium taxifolium* (Kunth) Govaerts (Lamiaceae), a Native Andean Species of Ecuador. Molecules.

[15] Gilardoni G., Montalván M., Ortiz M., Vinueza D., Montesinos J.V. (2020). The Flower Essential Oil of *Dalea mutisii* Kunth (Fabaceae) from Ecuador: Chemical, Enantioselective, and Olfactometric Analyses. Plants.

[16] Gilardoni G., Matute Y., Ramírez J. (2020). Chemical and Enantioselective Analysis of the Leaf Essential Oil from *Piper coruscans* Kunth (Piperaceae), a Costal and Amazonian Native Species of Ecuador. Plants.

[17] Tropicos.org. Missouri Botanical Garden. https://www.tropicos.org.

[18] Jorgensen P., Leon-Yanez S. (1999). Catalogue of the Vascular Plants of Ecuador.

[19] Jakupovic J., Zdero C., King R.M. (1995). Furanoeremophilanes from *Gynoxys* Species. Phytochemistry.

[20] Ordóñez P.E., Quave C.L., Reynolds W.F., Varughesea K.I., Berry B., Breena P.J., Malagón O., Smeltzer M.S., Compadre C.M. (2011). Sesquiterpene Lactones from *Gynoxys verrucosa* and their Anti-MRSA Activity. J. Ethnopharmacol..

[21] Ordóñez P.E., Sharma K.K., Bystrom L.M., Alas M.A., Enriquez R.G., Malagón O., Jones D.E., Guzman M.L., Compadre C.M. (2016). Dehydroleucodine, a Sesquiterpene Lactone from *Gynoxys verrucosa*, Demonstrates Cytotoxic Activity against Human Leukemia Cells. J. Nat. Prod..

[22] Catalano S., Cioni P.L., Menichini A., Bilia A.B., Morelli L., De Feo V. (1993). Kauranoid Diterpenes in *Gynoxys oleifolia*. Planta Med..

[23] Bohlmann F., Grenz M., Suwita A. (1977). Inhaltsstoffe aus *Gynoxys*- und *Pseudogynoxys*-arten. Phytochemistry.

[24] Zdero C., Bohlmann F., Robinson H., King R.M. (1980). Neue Furanoeremophilane aus *Gynoxys dielsiana*. Phytochemistry.

[25] Bohlmann F., Zdero C. (1979). Ein Neues Furanoeremophilon-derivat aus *Gynoxys psilophylla*. Phytochemistry.

[26] Hernández J., Rojas-Fermina L.B., Amaro-Luis J., Pouységu L., Quideau S., Usubillaga A. (2015). Chemical Composition of the Essential Oil of *Gynoxys meridana* from Mérida, Venezuela. Nat. Prod. Commun..

[27] Valarezo E., Aguilera-Sarmiento R., Meneses M.A., Morocho V. (2021). Study of Essential Oils from Leaves of Asteraceae Family Species *Ageratina dendroides* and *Gynoxys verrucose*. J. Essent. Oil-Bear. Plants.

[28] Babushok V.I., Linstrom P.J., Zenkevich I.G. (2011). Retention Indices for Frequently Reported Compounds of Plant Essential Oils. J. Phys. Chem. Ref. Data.

[29] Chisholm M.G., Wilson M.A., Gaskey G.M. (2003). Characterization of Aroma Volatiles in Key Lime Essential Oils (*Cirtrus aurantifolia* Swingle). Flavour Fragr. J..

[30] Elmore J.S., Nisyrios I., Mottram D.S. (2005). Analysis of the Headspace Aroma Compounds of Walnuts (*Juglans regia* L.). Flavour Fragr. J..

[31] Cozzani S., Muselli A., Desjobert J.-M., Bernardini A.-F., Tomi F., Casanova J. (2005). Chemical Composition of Essential Oil of *Teucrium polium* subsp. *capitatum* (L.) from Corsica. Flavour Fragr. J..

[32] Hachicha S.F., Skanji T., Barrek S., Ghrabi Z.G., Zarrouk H. (2007). Composition of the Essential Oil of *Teucrium ramosissimum* Desf. (Lamiaceae) from Tunisia. Flavour Fragr. J..

[33] Kundakovic T., Fokialakis N., Kovacevic N., Chinou I. (2007). Essential Oil Composition of *Achillea lingulata* and *A. umbellate*. Flavour Fragr. J..

[34] Saroglou V., Marin P.D., Rancic A., Veljic M., Skaltsa H. (2007). Composition and Antimicrobial Activity of the Essential Oil of Six *Hypericum* Species from Serbia. Biochem. Syst. Ecol..

[35] Muselli A., Rossi P.-G., Desjobert J.-M., Bernardini A.-F., Berti L., Costa J. (2007). Chemical Composition and Antibacterial Activity of *Otanthus maritimus* (L.) Hoffmanns. Link Essential Oils from Corsica. Flavour Fragr. J..

[36] Gonny M., Cavaleiro C., Salgueiro L., Casanova J. (2006). Analysis of *Juniperus communis* subsp. *alpina* Needle, Berry, Wood and Root Oils by Combination of GC, GC/MS and ^13^C-NMR. Flavour Fragr. J..

[37] Skocibusic M., Bezic N., Dunkic V. (2006). Phytochemical Composition and Antimicrobial Activities of the Essential Oils from *Satureja subspicata* Vis. Growing in Croatia. Food Chem..

[38] Stashenko E., Wiame H., Dassy S., Martinez J.R., Shibamoto T. (1995). Catalytic Transformation of Copaiba (*Copaifera officinalis*) Oil over Zeolite ZSM-5. J. High Res. Chromatogr..

[39] Mazzoni V., Tomi F., Casanova J. (1999). A Daucane-type Sesquiterpene from *Faucus carota* Seed Oil. Flavour Fragr. J..

[40] Martinez J., Rosa P.T.V., Menut C., Leydet A., Brat P., Pallet D., Meireles M.A.A. (2004). Valorization of Brazilian Vetiver (*Vetiveria zizanioides* (L.) Nash ex Small) Oil. J. Agric. Food Chem..

[41] Wong K.C., Lim T.B., Ali D.M.H. (2006). Essential Oil of *Homalomena sagittifolia* Jungh. Flavour Fragr. J..

[42] Adams R.P. (2007). Identification of Essential Oil Components by Gas Chromatography/Mass Spectrometry.

[43] Gil M.L., Jimenez J., Ocete M.A., Zarzuelo A., Cabo M.M. (1989). Comparative Study of Different Essential Oils of *Bupleurum gibraltaricum* Lamarck. Pharmazie.

[44] Falk A.A., Hagberg M.T., Lof A.E., Wigaeus-Hjelm E.M., Wang Z.P. (1990). Uptake, Distribution, and Elimination of alpha-Pinene in Man after Exposure by Inhalation. Scand. J. Work Environ. Health.

[45] Kose E.O., Deniz I.G., Sarikurkcu C., Aktas O., Yavuz M. (2010). Chemical Composition, Antimicrobial and Antioxidant Activities of the Essential Oils of *Sideritis erythrantha* Boiss. and Heldr. (var. *erythrantha* and var. *cedretorum* P.H. Davis) Endemic in Turkey. Food Chem. Toxicol..

[46] Rivas da Silva A.C., Lopes P.M., Barros de Azevedo M.M., Costa D.C., Alviano C.S., Alviano D.S. (2012). Biological Activities of alpha-Pinene and beta-Pinene Enantiomers. Molecules.

[47] Rodrigues K.A., Amorim L.V., Dias C.N., Moraes D.F., Carneiro S.M., Carvalho F.A. (2015). *Syzygium cumini* (L.) Skeels Essential Oil and Its Major Constituent alpha-Pinene Exhibit anti-Leishmania Activity through Immunomodulation in vitro. J. Ethnopharmacol..

[48] Govindarajan M., Rajeswary M., Hoti S.L., Bhattacharyya A., Benelli G. (2016). Eugenol, alpha-Pinene and beta-Caryophyllene from *Plectranthus barbatus* Essential Oil as Eco-friendly Larvicides against Malaria, Dengue and Japanese Encephalitis Mosquito Vectors. Parasitol. Res..

[49] Perry N.S., Houghton P.J., Theobald A., Jenner P., Perry E.K. (2000). In-vitro Inhibition of Human Erythrocyte Acetylcholinesterase by *Salvia lavandulaefolia* Essential Oil and Constituent Terpenes. J. Pharm. Pharmacol..

[50] Miyazawa M., Yamafuji C. (2005). Inhibition of Acetylcholinesterase Activity by Bicyclic Monoterpenoids. J. Agric. Food Chem..

[51] Montalván M., Peñafiel M.A., Ramírez J., Cumbicus N., Bec N., Larroque C., Bicchi C., Gilardoni G. (2019). Chemical Composition, Enantiomeric Distribution, and Sensory Evaluation of the Essential Oils Distilled from the Ecuadorian Species *Myrcianthes myrsinoides* (Kunth) Grifo and *Myrcia mollis* (Kunth) DC. (Myrtaceae). Plants.

[52] Espinosa S., Bec N., Larroque C., Ramírez J., Sgorbini B., Bicchi C., Gilardoni G. (2019). Chemical, Enantioselective, and Sensory Analysis of a Cholinesterase Inhibitor Essential Oil from *Coreopsis triloba* S.F. Blake (Asteraceae). Plants.

[53] Salehi B., Upadhyay S., Erdogan Orhan I., Kumar Jugran A., Jayaweera S.L.D., A. Dias D., Sharopov F., Taheri Y., Martins N., Baghalpour N. (2019). Therapeutic Potential of α- and β-Pinene: A Miracle Gift of Nature. Biomolecules.

[54] Kasuya H., Okada N., Kubohara M., Satou T., Masuo Y., Koike K. (2015). Expression of BDNF and TH mRNA in the Brain Following Inhaled Administration of alpha-Pinene. Phytother. Res..

[55] Satou T., Kasuya H., Maeda K., Koike K. (2014). Daily Inhalation of alpha-Pinene in Mice: Effects on Behavior and Organ Accumulation. Phytother. Res..

[56] Lima D.F., Brandão M.S., Moura J.B., Leitão J.M.R.S., Carvalho F.A.A., Miúra L.M.C.V., Leite J.R.S.A., Sousa D.P., Almeida F.R.C. (2012). Antinociceptive Activity of the Monoterpene α-Phellandrene in Rodents: Possible Mechanisms of Action. J. Pharm. Pharmacol..

[57] Piccinelli A.C., Santos J.A., Konkiewitz E.C., Oesterreich S.A., Nazari-Formagio A.S., Croda J., Ziff E.B., Leite-Kassuya C.A. (2014). Antihyperalgesic and Antidepressive Actions of (*R*)-(+)-Limonene, α-Phellandrene, and Essential Oil from *Schinus terebinthifolius* Fruits in a Neuropathic Pain Model. Nutr. Neurosci..

[58] Lin J.J., Lin J.H., Hsu S.C., Weng S.W., Huang Y.P., Tang N.Y., Lin J.G., Chung J.G. (2013). Alpha-phellandrene Promotes Immune Responses in Normal Mice Through Enhancing Macrophage Phagocytosis and Natural Killer Cell Activities. In Vivo.

[59] Lin J.J., Wu C.C., Hsu S.C., Weng S.W., Ma Y.S., Huang Y.P., Lin J.G., Chung J.G. (2015). Alpha-Phellandrene-Induced DNA Damage and Affect DNA Repair Protein Expression in WEHI-3 Murine Leukemia Cells In Vitro. Environ. Toxicol..

[60] Hsieh L.C., Hsieh S.L., Chen C.T., Chung J.G., Wang J.J., Wu C.C. (2015). Induction of α-Phellandrene on Autophagy in Human Liver Tumor Cells. Am. J. Chin. Med..

[61] Røstelien T., Borg-Karlson A.K., Fäldt J., Jacobsson U., Mustaparta H. (2000). The Plant Sesquiterpene Germacrene D Specifically Activates a Major Type of Antennal Receptor Neuron of the Tobacco Budworm Moth *Heliothis virescens*. Chem. Senses.

[62] Mozuraitis R., Stranden M., Ramirez M.I., Borg-Karlson A.K., Mustaparta H. (2002). (-)-Germacrene D Increases Attraction and Oviposition by the Tobacco Budworm Moth *Heliothis virescens*. Chem. Senses.

[63] Stranden M., Liblikas I., Koenig W.A., Almaas T.J., Borg-Karlson A.K., Mustaparta H. (2003). (–)-Germacrene D Receptor Neurones in Three Species of Heliothine Moths: Structure-activity Relationships. J. Comp. Physiol. A.

[64] Müller M., Buchbauer G. (2011). Essential Oil Components as Pheromones. A Review. Flavour Fragr. J..

[65] Van Den Dool H., Kratz P.D. (1963). A Generalization of the Retention Index System Including Linear Temperature Programmed Gas—Liquid Partition Chromatography. J. Chromatogr..

[66] Tissot E., Rochat S., Debonneville C., Chaintreau A. (2012). Rapid GC-FID Quantification Technique without Authentic Samples Using Predicted Response Factors. Flavour Fragr. J..

